# High-Efficiency and Low-Resistance Melt-Blown/Electrospun PLA Composites for Air Filtration

**DOI:** 10.3390/polym17030424

**Published:** 2025-02-06

**Authors:** Yongmei Guo, Mingzhu Wu, Xiaojian Ye, Shengchao Wei, Luming Huang, Hailing Guo

**Affiliations:** 1 Fujian Key Laboratory of Novel Functional Textile Fibers and Materials, Clothing and Design Faculty, Minjiang University, Fuzhou 350108, China; 2 State Key Laboratory of Heavy Oil Processing, College of Chemical Engineering, China University of Petroleum (East China), Qingdao 266580, China; 3 Fuzhou Chunhui Clothing Limited Company, Fuzhou 350108, China

**Keywords:** polylactic acid, melt blowing, electrospinning, filtration performance

## Abstract

Biodegradable polylactic acid (PLA) was used to fabricate nonwoven fabrics via the melt blowing process, followed by electrospinning to deposit a nanofiber membrane. This composite process yielded PLA melt-blown/electrospun composite materials with excellent filtration performance. The effects of the solution concentration and spinning duration on the composite structure and material performance were investigated. The optimal composite was produced using a 10 wt.% PLA spinning solution prepared with a solvent mixture of dichloromethane (DCM) and N, N-dimethylformamide (DMF) in a 75/25 weight ratio. The process parameters included a spinning duration of 5 h, 18 kV voltage, 1.5 mL/h flow rate, and 12 cm collection distance. The resulting composite achieved a filtration efficiency of 98.7%, a pressure drop of 142 Pa, an average pore size of 5 μm, and a contact angle of 138.7°. These results provided optimal process parameters for preparing PLA melt-blown/electrospun composite filtration materials. This study highlights the potential of hydrophobic PLA composites with high filtration efficiency and low air resistance as environmentally friendly alternatives to traditional non-degradable filtration materials.

## 1. Introduction

The increasing global emphasis on sustainability and environmental protection has intensified the demand for biodegradable filtration materials [1,2]. Conventional non-biodegradable materials, such as polypropylene and other petroleum-based polymers, dominate the filtration industry due to their excellent mechanical properties, high filtration efficiency, and cost-effectiveness. However, these materials present significant environmental challenges, as their long degradation times and reliance on fossil resources contribute to escalating plastic waste pollution and environmental degradation [3]. Additionally, the disposal of single-use filtration products, such as face masks and industrial filters, has become a critical concern, particularly during global crises like the COVID-19 pandemic, where their use surged dramatically [4].

Biodegradable filter materials have emerged as a potential solution to this problem. These materials can be decomposed into harmless small molecules by microorganisms in the natural environment, significantly reducing the burden on the ecosystem [5]. Among biodegradable filter materials, polylactic acid (PLA)-based composite materials have emerged as a promising solution to address these limitations. PLA, derived from renewable resources such as corn starch or sugarcane, offers biodegradability and reduced carbon emissions compared to traditional polymers [6,7]. In addition to its excellent biodegradability, PLA also has good mechanical properties and non-toxic degradation products, making it a promising alternative to petroleum-based plastics [8].

Various technologies for processing PLA materials have been developed and designed, including melt blending, solution blending, electrospinning, injection molding, blend spinning, nanocomposite technology, 3D printing, and bio-material blending [9,10,11,12,13,14]. Among these technologies, the specific structural properties of PLA materials make its production more promising through melt blowing and spinning processing technologies [15,16,17,18]. PLA possesses a low melting point and moderate melt viscosity, which facilitates its thermal processing and ensures stable flow behavior during the melt blowing process [9]. Additionally, its tunable rheological properties enable the formation of uniform fibers in both the melt blowing and electrospinning processes. PLA also exhibits high tensile strength and elastic modulus, characteristics attributed to its polymer chain structure, which support fiber stretching and shaping. These properties make it suitable for using the melt blowing and electrospinning techniques.

Melt blowing is a technique that utilizes high-temperature molten PLA material, combined with high-pressure air streams, to stretch the melt into fine fibers [18,19]. Melt blowing can generate fibers with diameters in the micrometer range, possessing a relatively large specific surface area [20,21]. However, PLA’s thermal stability is relatively poor, and during the melt blowing process, it is prone to degradation, resulting in a decrease in molecular weight, which negatively impacts the mechanical properties of the fibers [22]. Electrospinning is a technique that applies a high voltage to stretch the PLA solution or melt it into nanometer- or micrometer-sized fibers using an electric field [23,24]. This method allows for the production of finer and more uniform fibers compared to traditional spinning techniques. The resulting fibers possess a large specific surface area, offering enhanced adsorption, filtration, and tunability [25,26]. However, during the electrospinning process, factors such as solution properties and injection speed can influence the quality, diameter distribution, and structure of the fibers [27]. To maintain fiber uniformity and improve yield, extensive research and optimization of the process are required [28,29].

Melt blowing technology enables the rapid production of nonwoven materials with relatively coarse fiber diameters (typically ranging from 1 to 10 µm), characterized by high air permeability and robust mechanical strength. In contrast, the electrospinning process produces nanofibers with diameters typically below 1 µm, offering an ultrahigh specific surface area and a fine pore structure. These properties enhance the ability to capture microscopic particles and significantly improve filtration efficiency. Combining melt blowing and electrospinning technologies can optimize and complement the performance of PLA filtration composite materials [30]. The melt-blown layer provides mechanical strength and air permeability as a support layer for PLA composite materials, and the spinning layer enhances the capture ability of fine particles as a functional layer, thereby constructing a PLA composite structure with high filtration efficiency and low pressure drop [31,32]. Additionally, the melt-blown layer captures larger particles, while the spinning layer captures smaller particles. The composite material formed has a graded filtration mechanism, which improves the overall filtration performance. Importantly, PLA materials can be used in both melt blowing and electrospinning technologies to ensure that the composite material is sustainable and biocompatible while achieving efficient filtration. By electrospinning PLA nanofibers onto melt-blown nonwoven fabrics, the filtration efficiency is significantly enhanced. The electrospun nanofiber membrane effectively addresses the shortcomings of the melt-blown nonwoven fabric, such as insufficient strength [33]. By regulating the fiber structure, surface functionalization, and porosity of the PLA melt-blown/electrospun composite material, its overall performance can be improved, thereby broadening its potential for various practical applications [34,35].

Herein, PLA melt-blown/electrospun composite materials were fabricated using a hybrid melt blowing–electrospinning process. The effect of the melt-blowing temperature on the structure of the PLA nonwoven fabrics was investigated. At the optimized melt-blowing temperature, PLA melt-blown nonwoven fabrics with a well-formed fiber network and morphology were obtained. Based on these optimized melt-blown fabrics, electrospinning was employed to prepare the composite material. The impact of electrospinning process parameters, including the solution concentration and spinning duration, on the structure and performance of the composites was also explored.

## 2. Experimental

### 2.1. Materials

Polylactic acid (PLA, spinning grade) was provided by Zhejiang Hisun Biomaterials Co., Ltd. (Taizhou, China). Dichloromethane (DCM, AR) and N, N-dimethylformamide (DMF, AR) were acquired from Sinopharm Chemical Reagent Co., Ltd. (Shanghai, China). The PLA melt-blown nonwoven fabric was made in the laboratory.

### 2.2. Preparation Procedure of the Polylactic Acid Melt-Blown Nonwoven Fabric

First, the screw of the melt-blowing extrusion part was preheated, and then the metering pump, die head, blast and other parts were preheated after the temperature of the screw extrusion part was close to the set temperature. After all temperatures reached the set temperature, the metering pump, extruder, and pressure gauge were opened in sequence, and the dried PLA slice raw material was slowly added to the feed port. The feed port cover was immediately covered to prevent PLA from absorbing moisture and to keep the PLA raw material at a low moisture content for the greatest extent of time. After the PLA raw material was added, the melt-blown cloth that was produced initially was not collected and used to avoid other impurities remaining in the melt-blowing machine from affecting the PLA. When the melt-blown cloth produced had a stable performance and uniform cloth surface, the melt-blown cloth was wound and collected.

In this experiment, the spinneret aperture selected was 0.5 mm, the screw extruder speed was set to 7 r/min, the metering pump speed was 12 Hz, the mesh belt receiving speed was 3.8 m/min, the drum winding speed was 1.5 m/min, the receiving distance was 18 cm, the blower wind speed was 21 m^3^/h, and the hot air pressure was 1.6 MPa. The molten PLA at different melt-blowing temperatures was melt-blown into ultrafine fibers according to the set parameters, and was received on the surface of the mesh belt, adhering to each other to form a fiber web.

This experiment mainly studied and determined the effects of different melt-blowing temperature processes on PLA melt-blown nonwoven fabrics. The temperature gradient set in the experiment was 10 °C. Table 1 shows the names of the PLA melt-blown nonwoven fabrics prepared under different melt-blowing temperature conditions.

### 2.3. Preparation Procedure of the Polylactic Acid Melt-Blown/Electrospun Composites

**Figure 1:**
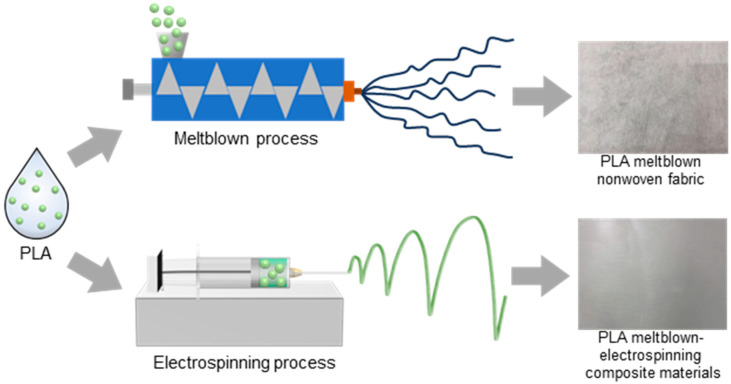
Preparation process of the PLA melt-blown/electrospun composite materials.

The experiment used single-needle electrospinning (in Figure 1). A disposable syringe with a capacity of 20 mL was used to load the PLA solution. The syringe was fixed on the injection pump at a set distance and connected to a high-voltage power supply. Then the PLA melt-blown nonwoven fabric was covered and fixed on the roller receiver. After the solution flow rate was set to 1.5 mL/h, with the receiving distance at 12 cm, the spinning voltage at 18 kV, and the spinning temperature at 25 °C, the power supply and voltage were turned on and the PLA electrospun fibers were sprayed on the PLA melt-blown nonwoven fabric to prepare a PLA melt-blown/electrospun composite material. This experiment studied and discussed the performance of the composite material by changing the concentration of the PLA solution and the spinning time. The corresponding preparation conditions are shown in Table 2 and Table 3, respectively.

The experimental setting used DCM/DMF with a mass ratio of 75/25 as the solvent. The concentration of the PLA solution was 2%, and the spinning time was 1 h. The PLA melt-blown/electrospun composite materials prepared under different spinning concentration solutions and different spinning time conditions can be seen in Table 2 and Table 3.

### 2.4. Characterization of the Filtration Efficiency and Resistance of PLA Melt-Blown Nonwoven Fabric

The mask filter material particle filtration efficiency tester was used to test the filtration performance of the PLA melt-blown nonwoven fabrics spun at different melt-blowing temperatures. After turning on the machine and the air pump, the filter material automatic tester software interface was entered to set the air volume to 85 LPM and then debug the concentration of the filtered particles. After the concentration of the particles was maintained at 25,000~35,000, sampling was performed. Next, after setting the number of tests, the sample was placed flat on the sample table and the test button was clicked to start the test. Each sample needed to be tested repeatedly in different areas, and finally the average value was obtained to obtain the filtration efficiency of the sample.

When testing the filtration efficiency, the resistance performance was also tested at the same time. It is worth noting that repeated tests needed to be performed in different areas of the sample to obtain the average value in order to obtain the resistance data of the sample.

### 2.5. Pore Size Characterization of the Polylactic Acid Melt-Blown Nonwoven Materials

The capillary pore size analyzer was used to test and study the pore size of spun PLA melt-blown nonwoven fabrics. Sample preparation was required before the experiment. A circular sample with a diameter of 25 mm was cut from the PLA melt-blown nonwoven fabric. The sample was naturally infiltrated with the infiltration liquid and placed flat on the sample table. After tightening the sample table screws, the air pump and the instrument was turned on. When the air pump pressure gauge was within the normal range, the pore size analyzer was connected to the computer software, where the experimental parameter range was set, and the test was clicked and started. After the sample test was complete, the machine automatically stopped and saved the data and charts for subsequent analysis.

### 2.6. Characterizations

A desktop scanning electron microscope (TM-4000, Hitachi High-Tech, Tokyo, Japan) was used to analyze the morphology of the composite materials. Before the experiment, the sample needed to be prepared. First, the conductive glue was flatly attached to the sample table. Then, the PLA melt-blown nonwoven fabric samples, prepared at different melt-blowing temperatures, were cut according to the specifications of 0.5 cm × 0.5 cm, and flatly attached to the sample table. The samples were gold-sprayed using an ion sputterer. After the sample was prepared, the electron microscope was turned on, and the sample was properly placed in the sample chamber. Finally, the computer control software was entered, and the scanning voltage mode was selected as 15 kV, Mode3, and a magnification was selected to observe the morphology of the composite material.

## 3. Results and Discussion

### 3.1. Morphology and Structure of the PLA Nonwoven Fabric

**Figure 2:**
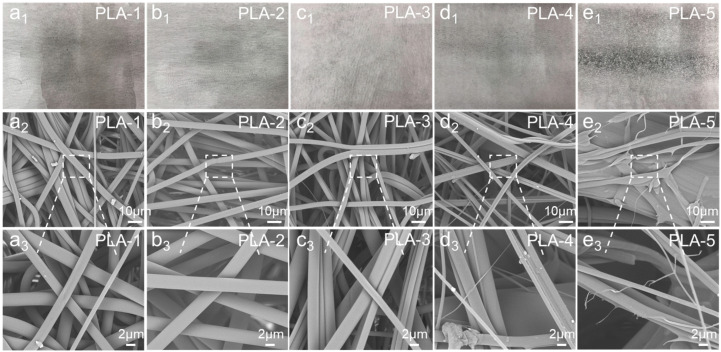
(**a1**–**e1**) Actual pictures of the PLA melt-blown nonwoven fabric with different melt-blowing temperatures. (**a2**–**e2**) Low magnification SEM images of the PLA melt-blown nonwoven fabric with different melt-blowing temperatures. (**a3**–**e3**) High magnification SEM images of the PLA melt-blown nonwoven fabric with different melt-blowing temperatures.

The melt-blowing temperature is one of the most critical process parameters in the fabrication of PLA melt-blown nonwoven fabrics, as it directly influences the viscosity and rheological behavior of the polymer melt. An insufficient melt-blowing temperature may result in poor melt flowability, hindering the drawing and formation of filaments. Conversely, excessively high temperatures may lead to thermal degradation of the PLA melt. Therefore, optimizing the melt-blowing temperature is essential for the successful preparation of PLA nonwoven fabrics using the melt blowing method. Figure 2(a1–e1) presents the physical appearance of the PLA melt-blown nonwoven fabrics produced at various melt-blowing temperatures. Macroscopic observations revealed that PLA-1 exhibited a small number of transparent crystalline particles at the center of the fabric surface. PLA-4 showed clear signs of hardening on the fabric surface, with brittle short fibers appearing at the edges. For PLA-5, complete embrittlement was evident, and the fabric readily fractured under slight force. In contrast, PLA-2 and PLA-3 displayed no significant defects upon macroscopic examination.

Figure 2(a2–e2,a3–e3) displays SEM images of the PLA melt-blown nonwoven fabrics prepared at different melt-blowing temperatures. As shown in Figure 2(a2–e2), PLA-1 demonstrated an overall satisfactory fiber formation with diameters ranging from 6 to 8 μm, exhibiting significant anisotropy. However, the fibers were inconsistent in thickness, and their surfaces were characterized by numerous particles. Combining these observations with the macroscopic analysis, it was inferred that although PLA was melted at the intended processing temperature for PLA-1, some crystalline granules remained partially unmelted. These granules were drawn into fine transparent particles during fiber formation, which deposited on the fabric surface, potentially affecting key properties such as hand feel, filtration efficiency, and airflow resistance. Figure 2(b2,b3) illustrates that PLA-2 fibers were uniform, smooth, and straight, with diameters of 9–10 μm, and were free of crystalline impurities. The fibrous web exhibited excellent formation quality under these conditions. In contrast, Figure 2(c2,c3) shows that fibers produced at the melt-blowing temperature for PLA-3 also had smooth surfaces with no apparent defects. However, the fibers exhibited a curled morphology and tended to adhere to one another, preventing the formation of discrete, straight fibers. This characteristic may contribute to higher airflow resistance in the resulting melt-blown fabric. Defects in the PLA nonwoven materials at high melt-blowing temperatures could be attributed to the fact that as the melt-blowing temperatures increase, the melt viscosity of PLA decreases, resulting in increased fiber fluidity at high temperatures. As a result, maintaining the stability of the fiber shape becomes challenging. In addition, increased melt-blowing temperatures led to insufficient cooling airflow, causing the fibers to remain in a viscous state before cooling and solidifying. This condition promotes fiber bonding and irregular molding.

**Figure 3:**
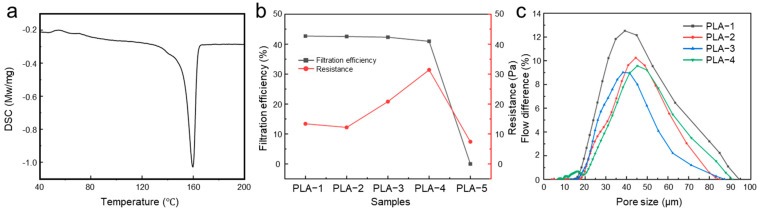
(**a**) DSC curve of the polylactic acid slice. (**b**) Filtration efficiency and resistance of the PLA nonwoven fabric with different melt-blowing temperatures. (**c**) Pore size distribution of the PLA melt-blown/electrospun composites with different (**a**) solution concentrations and (**b**) spinning times.

The DSC results of the PLA slice material is presented in Figure 3a. The results indicated that the melting point of the PLA material was approximately 159 °C. PLA is almost completely melted at around 165 °C, changing from a solid state to a molten state. The melting points of traditional polypropylene, polyester, and polyamide materials are generally between 200–260 °C. The low melting point of polylactic acid materials makes the energy consumption of its processing low, which is suitable for heat-sensitive equipment. In addition, the narrow melt-blowing processing temperature range facilitated the precise control of fiber diameter and was suitable for the production of fine fibers. Figure 3b demonstrates that the melt-blown temperature had little effect on the filtration efficiency of the PLA melt-blown nonwoven fabrics that successfully formed fibers and webs. However, it significantly impacted the resistance of the fiber web. This was because the melt-blowing temperature directly influences the fiber drawing and formation, which plays a crucial role in determining the fiber morphology and, consequently, the resistance performance of the melt-blown fabric. Excluding PLA-5, which exhibited severe brittleness and failed to form a proper web, PLA-2—featuring uniform, fine, and straight fibers with no apparent surface defects—exhibited the lowest resistance at only 12.17 Pa. In contrast, PLA-4, characterized by multiple fiber adhesions and noticeable clumps of defects, displayed the highest resistance, reaching 31.4 Pa.

Figure 3b presents the pore size distribution analysis of the PLA melt-blown nonwoven fabrics prepared at different melt-blowing temperatures. Due to severe brittleness and negligible hygroscopicity of the PLA-5 sample, it could not be wetted and thus was excluded from pore size analysis. To minimize the influence of the other variables on pore size distribution, all samples were tested under consistent size and base weight conditions. The results indicated that the pore size distributions of the four analyzable samples were relatively similar. PLA-1 and PLA-2 exhibited comparable fiber morphologies, with smooth, fine, and straight fibers of high uniformity. However, PLA-1 contained small residual crystalline grains from incomplete melting, resulting in a slightly smaller average pore size compared to PLA-2. At a melt-blowing temperature of 200 °C, PLA-3 fibers, though free of significant surface defects, displayed curling and inter-fiber adhesion, leading to a broader pore size distribution. The smallest pore size observed for PLA-3 was 4.7 μm. At 210 °C, PLA-4 fibers exhibited extensive inter-fiber adhesion, causing irregular fiber thickness, clumps, and poor uniformity. The fibers in PLA-4 were arranged chaotically, resulting in the widest pore size distribution among all samples.

### 3.2. Morphology and Structure of the Polylactic Acid Melt-Blown/Electrospun Composites

**Figure 4:**
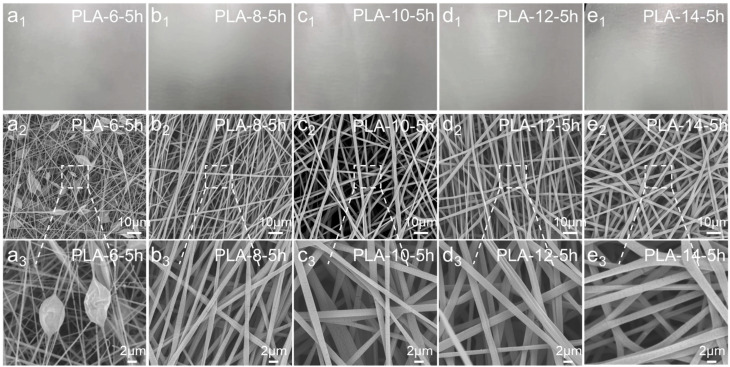
(**a1**–**e1**) Actual pictures of the PLA melt-blown/electrospun composite materials with different solution concentrations. (**a2**–**e2**) Low magnification SEM images of PLA melt-blown/electrospun composites with different solution concentrations. (**a3**–**e3**) High magnification SEM images of PLA melt-blown/electrospun composites with different solution concentrations.

Figure 4(a1–e1) shows the actual images of PLA melt-blown/electrospun composite materials spun for 5 h with different concentrations of the spinning solution under the same other conditions. It was observed that when the spinning time and other conditions were exactly the same, the macroscopic physical aspects of the melt-blown/electrospun composite materials spun by spinning solutions of different concentrations were not much different. However, it can be observed from Figure 4(e1) that white spots appeared on the surface of the PLA-14-5h sample. This is because the solution concentration of the sample was relatively high. During the spinning process, the solvent evaporated quickly, and PLA could not be stretched into filaments. Small droplets were formed at the needle tip to block the needle. As the injection pump continued to advance and the high-voltage power supply was used, the small droplets were ejected and received by the base cloth, leaving white spots on the sample surface.

**Figure 5:**
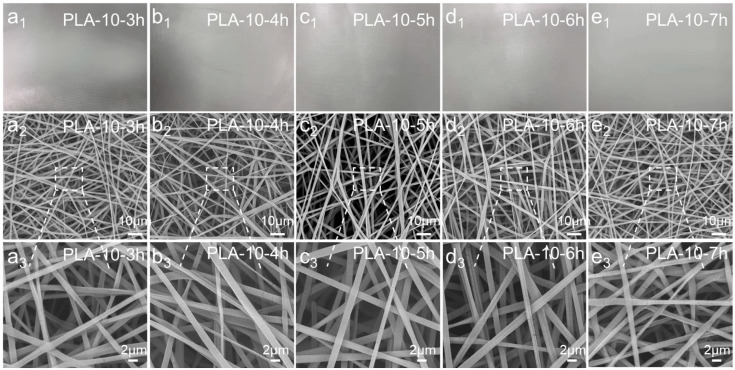
(**a1**–**e1**) Actual pictures of PLA melt-blown/electrospun composite materials with different spinning times. (**a2**–**e2**) Low magnification SEM images of PLA melt-blown/electrospun composites with different spinning times. (**a3**–**e3**) High magnification SEM images of PLA melt-blown/electrospun composites with different spinning times.

Figure 4(a2–e2) shows the SEM images of the melt-blown/electrospun composites spun for 5 h with different concentrations of the spinning solution under otherwise identical conditions. It was observed from the figure that in a solution with a DCM/DMF mass ratio of 75/25 and a concentration of 8 wt.%, 10 wt.%, 12 wt.%, and 14 wt.%, PLA can spin uniform and isotropic products and fibers with significant heterosexuality. But, when the concentration was 6 wt.%, a large number of beaded structures appeared in the fibers, and some fibers broke. This is because the concentration of the spinning solution was too low, causing the PLA to break before being received by the collection device, resulting in fiber breakage, beading, and other phenomena. From the PLA-8-5h in Figure 5(b2,b3) it was found that although the diameter of a single fiber electrospun at this concentration was the smallest among the four concentrations, two or three fibers were adherent and entangled with each other in a large area when the fiber was synthesized. The electrospun fibers of PLA-10-5h had distinct roots and showed obvious anisotropy. The fiber surface in PLA-12-5h also showed a flat and concave fiber morphology, and some fibers suffered from the same fiber adhesion as PLA-8-5h. In the PLA-14-5h sample, the fibers at this concentration were uniform and thin, but the fiber diameter was too thick, reaching two to three times the fiber diameter of the PLA-10-5h sample.

This study found that as the concentration of the spinning solution increased, the fiber diameter gradually increased. When observing the SEM images at large magnification, it was found that many small cracks appeared on the surface of the fiber. This is because when the electron microscope observation magnification was larger, the electron microscope filament was closer to the sample, and the filament had a higher temperature. Excessive temperature of the filament had an impact on the sample, causing small cracks to appear on the surface of the fiber.

Figure 5 depicts the morphologies of the melt-blown/electrospun composite materials prepared from a 10 wt.% PLA solution at different spinning times. By observing the SEM images, it was found that the change in spinning time did not have much effect on the fiber morphology of the sample. When other conditions remained unchanged, the density of the fiber arrangement in the web increased significantly with the increase in spinning time. The spinning time of the electrospinning mainly affected the filtration efficiency and other properties of the composite material by increasing the macroscopic thickness of the melt-blown/electrospun composite material and the density of the microscopic fiber arrangement.

### 3.3. Filtration Efficiency of the Polylactic Acid Melt-Blown/Electrospun Composite Materials

**Figure 6:**
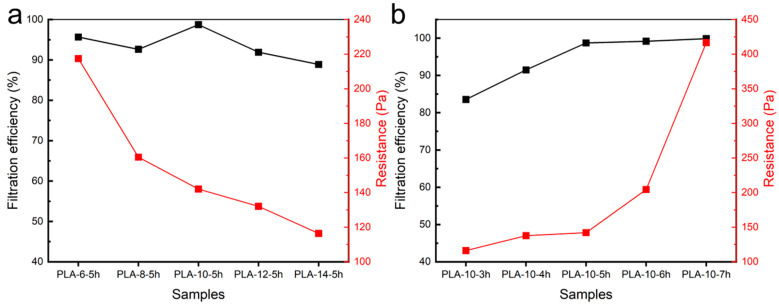
Filtration efficiency and resistance of the PLA melt-blown/electrospun composites with (**a**) different solution concentration and (**b**) spinning times.

Figure 6a exhibit the filtration efficiency and resistance data of the PLA melt-blown/electrospun composite materials under different solution concentrations. As can be seen from Figure 6a, when other conditions remained unchanged, with the increase in the solution concentration, the filtration efficiency of the PLA melt-blown/electrospun composite materials generally showed a trend of increasing first and then decreasing. Correspondingly, the resistance decreased with the increase in solution concentration. This may be due to the fact that when the solution concentration increased, the fiber morphology changed, the fiber diameter gradually increased, and the filtration efficiency and resistance of the sample changed with the change in fiber morphology. As seen, the filtration efficiency of the PLA melt-blown/electrospun composite materials with the five concentrations of 6 wt.%, 8 wt.%, 10 wt.%, 12 wt.%, and 14 wt.% reached more than 88%, and the filtration efficiency of the composite material with a concentration of 10 wt.% reached 98.7%. Compared with the homemade PLA melt-blown cloth with a filtration efficiency of only 42%, the filtration efficiency of the five composite materials was greatly improved, all of which were increased by more than 45%.

Figure 6b depicts the filtration efficiency and resistance of the PLA melt-blown/electrospun composites prepared at different spinning durations. It was observed that, at a spinning solution concentration of 10 wt.%, both filtration efficiency and resistance of the composites increased with a prolonged spinning time. Specifically, at a spinning duration of 5 h, the PLA-10-5h composite exhibited a filtration efficiency of 98.7% with a resistance of only 142 Pa. Compared to PLA-10-4h, the filtration efficiency showed a significant improvement, while the resistance remained nearly unchanged. However, further extending the spinning duration to 6 and 7 h resulted in marginal improvements in filtration efficiency, reaching 99.18% and 99.87%, respectively, but it was accompanied by a substantial increase in resistance to 204 Pa and 416 Pa, respectively, leading to a sharp decline in air permeability.

### 3.4. Pore Characteristic of the Polylactic Acid Melt-Blown/Electrospun Composites

**Figure 7:**
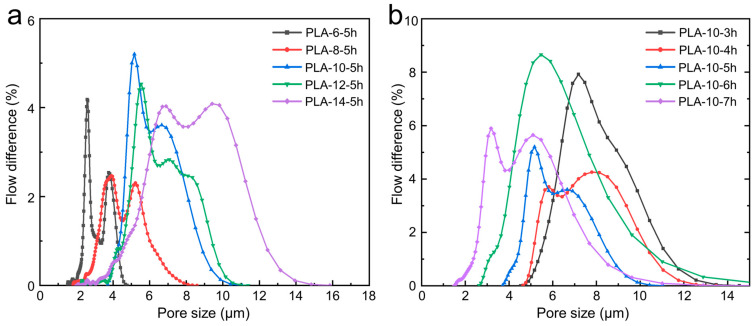
Pore size distribution of the PLA melt-blown/electrospun composites with different (**a**) solution concentrations and (**b**) spinning times.

By analyzing Figure 7a, and Table 4, it is evident that the solution concentration significantly affected the pore size distribution and uniformity of the PLA melt-blown/electrospun composites. As the solution concentration increased, the average pore size of the composites gradually enlarged, and the pore size distribution range broadened. This was attributed to the fact that higher solution concentrations lead to larger fiber diameters, resulting in an increase in the pore size of the fibrous network. Additionally, the pore size distribution of the samples with different concentrations was not uniform. This inconsistency may be due to the use of a single-needle electrospinning setup in this study, which tends to produce composites with a thicker center and thinner edges. When preparing samples for pore size testing, random sections of the composite were cut for analysis, potentially contributing to the observed non-uniform pore size distribution.

Figure 7b and Table 5 shows the pore distribution of the PLA melt-blown/electrospun composite materials with different spinning duration. The spinning duration has a significant impact on the average pore size of PLA melt-blown/electrospun composites. As the spinning duration increases, the pore size of the samples gradually decreases. Observations from Figure 7b indicate that the range of pore size distribution remains relatively constant with increasing spinning duration. The pore size distribution curve visually reflects the uniformity of the sample’s pore structure—the more irregular the curve, the less uniform the pore distribution. This non-uniformity directly affects the filtration efficiency and resistance performance of the sample.

### 3.5. Wettability of the Polylactic Acid Melt-Blown/Electrospun Composites

**Figure 8:**
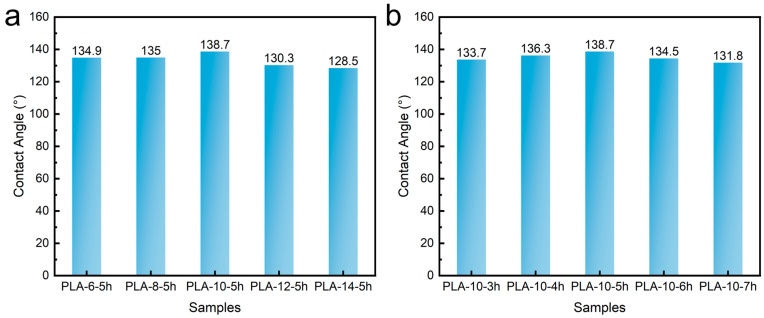
Contact angle of the PLA melt-blown/electrospun composites with different (**a**) solution concentrations and (**b**) spinning times.

Under high humidity conditions, poor hydrophobicity of the filtration materials can result in the material being easily wetted by air moisture. This leads to a sharp decline in filtration performance and a significant increase in pressure drop, severely compromising the efficiency of the filtration material. Therefore, wettability is one of the critical performance indicators for filtration materials. Figure 8a shows the contact angles of the PLA melt-blown/electrospun composites prepared with different solution concentrations. As seen, the contact angles of all the PLA melt-blown/electrospun composites in this study exceeded 128°, indicating that all PLA melt-blown/electrospun composite materials were hydrophobic. With increasing solution concentration, the contact angle initially increased and then decreased, though the overall variation remained minimal. Among them, PLA-10-5h exhibited the highest contact angle of 138.7°. The spun fibers made from conventional polypropylene and polyester materials are usually in the range of 90–110° [36], which is much lower than that of the PLA melt-blown/electrospun composites produced in this study, suggesting that the PLA melt-blown/electrospun composites have superior hydrophobicity. This study confirmed the ability to modulate the fiber structure through the combination of the melt blowing and spinning processes, enabling the efficient fabrication of superhydrophobic fiber membranes.

Figure 8b shows the contact angles of the PLA melt-blown/electrospun composites prepared at different spinning durations. According to the figure, all samples exhibited contact angles exceeding 130°, confirming their hydrophobic nature. The contact angles increased initially and then decreased with extended spinning time, though the overall variation was relatively small. The sample with the best hydrophobic performance remains PLA-10-5h, with a maximum contact angle of 138.7°.

## 4. Conclusions

In conclusion, biodegradable PLA was used to prepare the melt-blown/electrospun composite filter materials. The structural properties of the composite materials were optimized by changing the melt-blowing temperature, electrospinning solution concentration, and spinning time. The melt-blowing temperature was critical for producing PLA nonwoven fabrics. Low temperatures resulted in the incomplete melting of PLA, leaving fine transparent grains on the fabric surface, while excessively high temperatures caused thermal degradation, leading to brittle fabrics. The solution concentration significantly impacted the morphology of the electrospun fibers, directly affecting the composite material’s properties. With the increase in solution concentration, the fiber diameter and average pore size gradually increased, the resistance became smaller and smaller, and the filtration efficiency and contact angle of the composite materials showed a trend of increasing first and then decreasing. Spinning time had minimal impact on the fiber morphology but directly affected the filtration efficiency and resistance. Longer spinning times enhanced the filtration efficiency and resistance, reduced the average pore size, and caused the contact angle to first increase and then decrease. Among the tested samples, the composite material prepared with a 10 wt.% PLA solution and a spinning time of 5 h showed the best performance, achieving a filtration efficiency of 98.7%, a resistance of 142 Pa, an average pore size of 5 μm, and a contact angle of 138.7°.

## Figures and Tables

**Table 1:** PLA melt-blown nonwoven fabrics prepared at different solution concentrations.

Samples	Screw Temperature/°C					Melt Temperature/°C	Hot Air Temperature/°C
	Zone 1	Zone 2	Zone 3	Zone 4	Zone 5		
PLA-1	160	180	180	180	180	180	230
PLA-2	160	190	190	190	190	190	230
PLA-3	160	200	200	200	200	200	230
PLA-4	160	210	210	210	210	210	230
PLA-5	160	220	220	220	220	220	230

**Table 2:** PLA melt-blown/electrospun composites prepared at different solution concentrations.

Samples	Solution Concentration	Spinning Time
PLA-6-5h	6 wt.%	5 h
PLA-8-5h	8 wt.%	5 h
PLA-10-5h	10 wt.%	5 h
PLA-12-5h	12 wt.%	5 h
PLA-14-5h	14 wt.%	5 h

**Table 3:** PLA melt-blown/electrospun composites prepared at different spinning times.

Samples	Concentration of Solution	Spinning Time
PLA-10-3h	10 wt.%	3 h
PLA-10-4h	10 wt.%	4 h
PLA-10-5h	10 wt.%	5 h
PLA-10-6h	10 wt.%	6 h
PLA-10-7h	10 wt.%	7 h

**Table 4:** Pore size of the PLA melt-blown/electrospun composites with different solution concentrations.

Samples	Maximum Pore Size/μm	Minimum Pore Size/μm	Average Pore Size/μm
PLA-6-5h	4.407	1.582	2.647
PLA-8-5h	7.652	1.924	3.912
PLA-10-5h	8.504	2.609	5.027
PLA-12-5h	9.792	2.652	5.776
PLA-14-5h	11.810	2.559	6.696

**Table 5:** Pore size of the PLA melt-blown/electrospun composites with different spinning times.

Samples	Maximum Pore Size/μm	Minimum Pore Size/μm	Average Pore Size/μm
PLA-10-3h	10.4268	2.436	6.910
PLA-10-4h	9.868	2.895	5.642
PLA-10-5h	8.504	2.609	5.027
PLA-10-6h	9.615	1.857	4.777
PLA-10-7h	7.672	1.247	3.468

## Data Availability

The original contributions presented in this study are included in the article. Further inquiries can be directed to the corresponding authors.
